# Leakage of Fluorescein Angiography Secondary to Rhegmatogenous Retinal Detachment: A Case Report

**DOI:** 10.7759/cureus.32763

**Published:** 2022-12-21

**Authors:** Ataa Rajeh, Hashem Abu Serhan, Abdulrahman Alfakir, Mohammad Askar, Amal Kassom

**Affiliations:** 1 Ophthalmology Department, Jordan Finland Modern Hospital, Amman, JOR; 2 Ophthalmology Department, Hamad Medical Corporation, Doha, QAT; 3 Ophthalmology Department, Medical City Polyclinic, Muscat, OMN; 4 Ophthalmology Department, Damascus University, Damascus, SYR; 5 Ophthalmology Department, Faculty of Medicine, Damascus University, Damascus, SYR

**Keywords:** fundus examination, contact lens, rhegmatogenous detachment, retinal detachment, fluorescence angiography

## Abstract

The identification and evaluation of leaking areas in FA pictures are essential steps in the diagnostic process as well as the treatment and management of a variety of choroidal and retinal illnesses. We reported a case of rhegmatogenous retinal detachment in a seven-year-old boy with positive leakage on FA, which confused the presentation. A thorough contact lens examination and scleral indentation are mandatory to reach the diagnosis easily.

## Introduction

The detection and assessment of areas of leakage in fluorescence angiography (FA) images are crucial for both the diagnosis and management of different choroidal and retinal diseases. Fluorescein leakage can occur due to attenuated blood vessels such as retinal neovascularization (NVE), choroidal neovascularization (CNV), or a detached retinal pigment epithelium (RPE) that no longer prevents fluorescein leakage from the choroid [1]. FA leakage is characterized by early hyperfluorescence, increasing with time in both area and intensity, and blurring of the vessel's margins [1]. It occurs as a result of two main mechanisms: dysfunction of existing vascular endothelial tight junctions as seen in diabetic retinopathy (DR), cystoid macular edema (CME), branch and central retinal vein occlusion or due to the primary absence of vascular endothelial tight junctions which seen in CNVs, Coats disease, or Behcet׳s disease [2]. We report fluorescein leakage secondary to rhegmatogenous retinal detachment (RRD) in a seven-year-old child and explain how this association made confusion to reach the diagnosis.

## Case presentation

**Figure 1:**
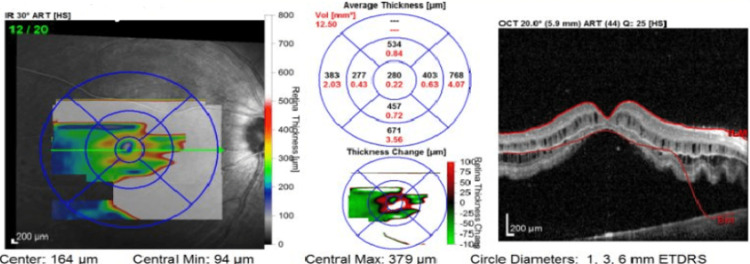
Optical coherence tomography of the right eye showing the detachment reached the macula

A seven-year-old male presented to the ophthalmology outpatient clinic complaining of sudden decreased vision in the right eye for nine days. No past history of photopsia or ocular trauma was reported. An ophthalmic examination of the right eye showed that the best-corrected visual acuity (BCVA) is 0.1. Left eye BCVA is 1. On fundus examination, the presence of pigment cells in the anterior vitreous, white temporal lesions floating in the vitreous cavity, and shallow inferior RRD involving the macula were observed. The left eye exam was unremarkable except for temporal white lesions involving the vitreous. No retinal breaks or telangiectasia were detected. Optical coherence tomography (OCT) of the right eye showed macula-off RD (Figure 1).

**Figure 2:**
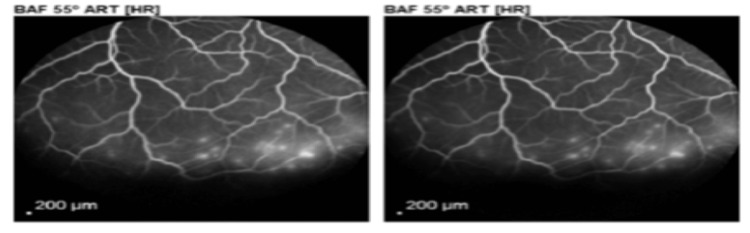
Fluorescein angiography of the right eye showing fluorescein leakage in the inferior detached retina

Even though the border of detachment was convex, the gravity dependency of fluid and the presence of smoothness or corrugations of the retinal surface couldn’t be judged well due to the shallowness of the detachment. B scan - to rule out posterior scleritis - showed unremarkable findings. To rule out the exudative RD, FA was ordered and showed retinal vascular leakage corresponding to the inferior area of detachment (Figure 2).

**Figure 3:**
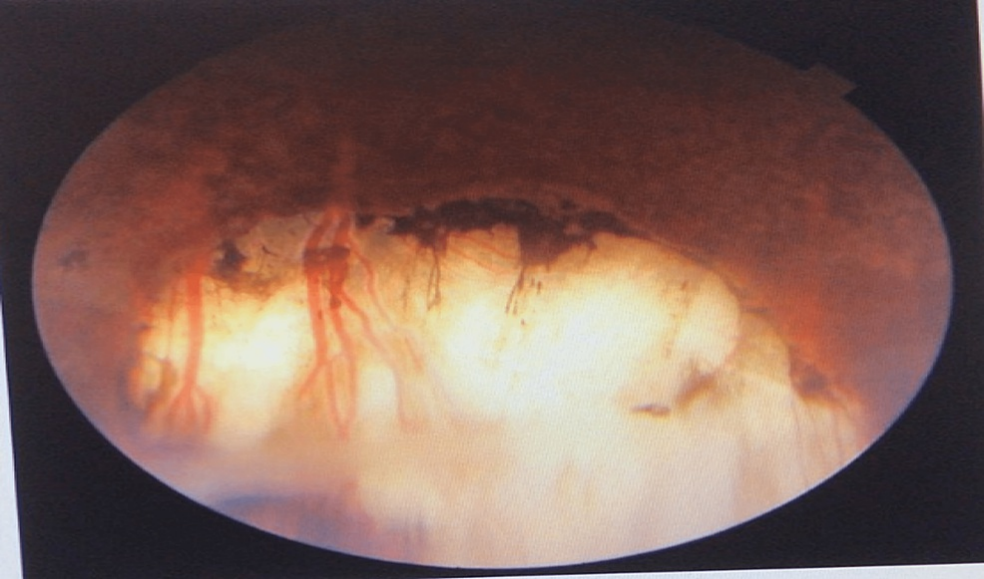
Right inferior retinal dialysis surrounded with cryotherapy marks after management with buckling surgery

Therefore, a vasculitis work-up was ordered and showed unremarkable findings. Contact lens fundus examination showed inferior dialysis from 5.00 to 7.00 clockwise, which was managed with buckling surgery (Figure 3). The patient was on regular follow-up for two years post-surgery with a stable examination.

**Figure 4:**
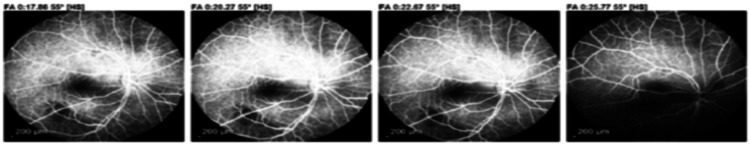
Fluorescein angiography of the right eye after buckling surgery showing the disappearance of fluorescein leakage

On follow-up examination, BCVA was improved to 0.4, the retina was flat and the leakage on repeating FA after eight months was absent (Figure 4).

## Discussion

This case report followed the CARE checklist for case reports guidelines [3]. RRD affects approximately about one in 10,000 of the population annually. In 10% of cases, bilateral eye involvement is seen. It is not uncommon in children, ranging from 3% to 12% of all patients suffering from RRD [4]. Tolentino et al.'s preliminary study on five patients with RRD reported the presence of leakage in FA secondary to RRD. The dye leakage was observed in longstanding RDs (>6 months) [5]. In addition, persistent leakage of fluorescein from the capillaries of the optic disc and posterior pole retinal vessels several months after reattachment surgery was noted. They also reported leakage in FA along the edge of retinal tears. Their size varied, but most were one disc in diameter or larger. Subclinical RRD around the tear was present in most of the cases [5]. Despite the presence of pigmented cells in the anterior vitreous in our patient, the presence of fluorescence leakage along vessels made confusion toward the possibility of vasculitis association.

Missed retinal breaks in RRD are responsible for almost 65% of the cases of failed RD surgery [6] leading to recurrent retinal detachments. Contact lens fundus examination with sclera indentation is crucial to avoid missing retinal breaks. Although our case may be considered a simple association between RRD and fluorescence leakage, we aim to shine on the importance of doing a thorough fundus exam with scleral indentation and contact lens [7].

## Conclusions

The detection and assessment of areas of leakage in FA images are crucial for the management of different choroidal and retinal diseases. There are many causes of leakage in FA. However, ophthalmologists should consider rhegmatogenous retinal detachment as one of the differentials of leakage in FA. Meticulous fundus examination with the appropriate lens should be done for all patients with visual loss complaints before running into extensive investigations.

## References

[1] Young CW (1979). Interpretation of fundus fluorescein angiography. Archives of Ophthalmology.

[2] Chams H, Mohtasham N, Davatchi F, Shahram F, Naji A, Aalami Harandi Z, Karimi N (2015). Ophthalmic findings in Behcet׳s disease: cases without apparent ocular signs. J Curr Ophthalmol.

[3] Gagnier JJ, Kienle G, Altman DG, Moher D, Sox H, Riley D (2013). The CARE guidelines: consensus-based clinical case reporting guideline development. J Med Case Rep.

[4] Fivgas GD, Capone A Jr (2001). Pediatric rhegmatogenous retinal detachment. Retina.

[5] Tolentino FI, Lapus JV, Novalis G, Trempe CL, Gutow GS, Ahmad A (1976). Fluorescein angiography of degenerative lesions of the peripheral fundus and rhegmatogenous retinal detachment. Int Ophthalmol Clin.

[6] Takkar B, Azad S, Shashni A, Pujari A, Bhatia I, Azad R (2016). Missed retinal breaks in rhegmatogenous retinal detachment. Int J Ophthalmol.

[7] Rahman R, Murray CD, Stephenson J (2013). Risk factors for iatrogenic retinal breaks induced by separation of posterior hyaloid face during 23-gauge pars plana vitrectomy. Eye (Lond).

